# What Does Biostatistics Mean To Us

**DOI:** 10.4103/0973-1229.27608

**Published:** 2006

**Authors:** Vance W. Berger, J. Rosser Matthews

**Affiliations:** * National Cancer Institute Bethesda, MD, USA *bergerv@mail.nih.gov*; ** Virginia Commonwealth University, USA *rmatthews@widomaker.com*

**Keywords:** *Biostatistics*, *Intuition*, *Controlled Studies*, *Randomization*, *Allocation Concealment*, *Masking*, *Type I and Type II Errors*, *Null Hypothesis*, *Bias*

## Abstract

It is human nature to try to recognize patterns and to make sense of that which we observe. Unfortunately, our intuition is often wrong, and so there is a need to impose some objectivity on the methods by which observations are converted into knowledge. One definition of biostatistics could be precisely this, the rigorous and objective conversion of medical and/or biological observations into knowledge. Both consumers of biostatistical principles and biostatisticians themselves vary in the extent to which they recognize the need to continue the improvement. Some may not recognize the need for (some or all of) the methods that have already been developed; others may accept these as they find them completely sufficient; still others recognize both the value and the shortcomings of these methods, and seek to develop even better methods to ensure that future medical conclusions are less subject to biases than current ones are.

## Introduction

### Two Classic Examples And A Definition

With or without biostatistical principles, it is human nature to try to convert observations into knowledge. It is also human nature to try to recognize patterns and make sense of observations. So, biostatistics could be defined initially as the descriptive enterprise of observing patterns in biomedical data. However, we argue that this is too broad a definition because observations often mislead and our intuitions are often wrong. Thus, we define it as follows:

*Biostatistics is the discipline concerned with how we* ought *to make decisions when analyzing biomedical data. It is the evolving discipline concerned with formulating explicit rules to compensate both for the fallibility of human intuition in general and for biases in study design in particular.*

To see why we need such a discipline, consider the example of having observed a long run of heads when a fair coin is tossed. Many would bet on tails next, arguing that it is due. The more enlightened would recognize the independence of the tosses, and how this translates into the inability of the past tosses to predict future ones. The even more enlightened would recognize the ease with which the word “fair” may be attached to a coin, whether or not the coin actually is fair. These individuals would use the past data to test the hypothesis that the coin is fair, and may find that in fact it comes up heads with a probability much larger than 50%; so they would bet heads next, and on every future toss of this coin.

Another classic example of fallible intuition concerns the famous question, “What is the probability that both children [in a family] are boys given that we know at least one of them is a boy?” If the probabilities of a boy and of a girl are 50% each, and if the genders of the two children are independent of each other, then one could argue that the first is a boy and the second has a 50% chance either way, so the answer is 50%. This answer confuses the question asked with another similar question, specifically, “What is the probability that both children are boys given that we know that the first one is a boy?” The second question excludes two of the *a priori* possible arrangements, GB and GG, leaving only BB and BG, each equally likely. So, 50% is the answer. Our original question did not exclude GB, however, and rather excluded only GG. The remaining possibilities are BB, BG, and GG, all equally likely, and the answer is 33%. Even this answer is not necessarily correct, however, because it is still based on two assumptions, specifically, that boys and girls are equally likely and that the genders of consecutive children are independent. The veracity of these assumptions was tested by Senn (2003, page 6), who examined a data set consisting of 6089 families with at least one child. Of these families, 2444 had exactly two children, with two boys in 582, two girls in 530, and one of each gender in 1332. Based on this data set, the estimate of the probability we seek is 1332/(1332+582)=70%, which is reasonably close to our theoretical 2/3.

These two classic examples have analogues in biostatistics, which we will consider in the next section. We will then discuss the necessity, but not the sufficiency, of current biostatistical principles. That is, the methods that are currently available do control the biases they were meant to control, but it is impossible to anticipate every bias that can occur, and to enumerate them. There will always be a need for diligence in checking that nothing unexpected goes wrong. When something amiss is found, one will need to use novel methods for dealing with this problem, and to salvage reliable comparisons.

## Classic Examples Applied To Medical Data

For the first example, tossing a “fair” coin, consider an unmasked randomized clinical trial using the popular and frequently used permuted blocks method of randomization. Suppose further that the block size is two (meaning that the first two patients constitute a block, as do the next two, and so on, and each treatment is allocated to one patient per block). Finally, suppose that the patient population is sufficiently heterogeneous so as to allow for better responders and worse responders. What would one conclude upon observing a long run of “healthier” patients being assigned to the active treatment group, and a long run of “sicker” patients being assigned to the control group? Is the control group due when the next “healthier” patient is to be enrolled? Is the active treatment due when the next “sicker” patient is to be enrolled? Does the process have any memory at all? Are the long runs in some way informative?

It is common knowledge that in a two-arm randomized clinical trial with 1:1 allocation to the two groups, each participant has the same chance of receiving either treatment. That is, until one really thinks about it. In our hypothetical unmasked trial with blocks of size two, the investigator will know the identity of every second allocation before a patient is selected to receive it. That is, if the first patient receives the control, then the next patient must necessarily receive the active treatment, and vice versa. Moreover, this pattern will repeat for each block, so the identity of the 2^nd^, 4^th^, 6^th^, and every other even-numbered allocation can be predicted based on the one preceding it (they will be opposites).

In such a case, it is certainly possible for the investigator to enroll “average” patients for the odd-numbered (unpredictable) accession numbers, “healthier” patients when it is known that the allocation will be to the active treatment, and “sicker” patients when it is known that the allocation will be to the control group (Berger, 2005a). This can be accomplished by judicious use of discretion in denying enrollment to patients who are not “suitable” for the treatment due to be assigned next, or by projecting doubts onto patients regarding the trial being in their best interest. It is even possible to defer the enrollment of a patient until the “matching” treatment is due to be assigned. Moreover, there is evidence that such subversion has actually occurred in many randomized clinical trials (Berger, 2005a; Berger and Weinstein, 2004).

If this type of subversion occurs, then clearly the metaphorical coin is not a fair one. There would be a systematic tendency for healthier patients to receive the active treatment and for sicker patients to receive the control treatment, a clear violation of the idea that each patient has an equal chance to receive each treatment. Moreover, this pattern observed in the past would be suggestive of the pattern continuing in the future (within the same trial); there would be no compensation, nor is there independence suggesting a lack of memory of the process.

In one formulation of our second example, we tried to determine the probability that both children are boys given that we know that at least one is a boy. This question was analyzed based on the assumption that each birth was independent. More specifically, as in the example of tossing a fair coin, the fact that the first child turned out to be a boy did not affect the probability that the second child would be a boy—which would still be 50%. In general, it is easy to make a sweeping claim that two processes operate independently, without recognizing either the subtlety or the context-dependent nature of the concept of independence. For example, everyone knows that height and weight are dependent. But is this true if the variation is with respect not to individuals, but rather to measurement times for a given individual? Here, the two would actually be independent. So the same two variables can be dependent or independent depending on the context.

Likewise, caution may be needed in concluding that two diseases are dependent. Consider observing, in a hospital setting, that those patients with cancer tend to present with heart disease less often than do those patients without cancer. On the surface, this might suggest that one disease protects against the other. However, suppose we represented the presence of these two diseases in a 2×2 contingency table thus:

**Table 1:** 2×2 Contingency Table

	Cancer	No Cancer
Heart Disease	(1,1)	(0,1)
No Heart Disease	(1,0)	(0,0)

In this notation “0” represents the absence of disease and “1” represents the presence of disease with the 1^st^ space in the parenthesis reserved for cancer and the second space reserved for heart disease. Then, this table would represent all possible outcomes (or “contingencies”) by which heart and cancer patients could be present in the hospital setting. Although there are reasons other than cancer and heart disease for being admitted to the hospital, for simplicity assume that these were the only two reasons to be admitted. In such a case, one would see three types of patients in the hospital, including (1,1) patients with both cancer and heart disease; (0,1) patients with heart disease but not cancer; and (1,0) patients with cancer but not heart disease. One would not see the (0,0) patients with neither disease in the hospital, because these would not be hospitalized.

Now suppose that cancer and heart disease are both independent and the chance of having or not having each disease is equally likely, so that the three types of patients that could be seen in the hospital, (0,1), (1,0), and (1,1), would be equally likely. From the table, it is easy to see that, among this hospital population, the probability of heart disease is 50% for those patients with cancer [half of the patients would be in cell (1,0) and half in cell (1,1)] and 100% for those patients without cancer [the only cell being counted is (0,1), and 100% of the patients in that cell have heart disease by definition]. On this analysis, the two diseases would appear to be negatively correlated, or that one is protective for the other. However, this is merely an artifact of the missing (0,0) cell, corresponding to patients with neither cancer nor heart disease (Berkson, 1946).

## Assisting Intuition: Controlled Studies, Randomization, Allocation Concealment And Masking

Recognition that our intuitions need to be supplemented, and that data can mislead, has created the necessity for a science that concerns itself with distinguishing valid inferences from spurious ones. This has led to the widespread use of improvements such as *controlled studies, randomization, allocation concealment, and masking*.

To see why these are important, consider evaluating the efficacy of an elective surgery. The most obvious way to do this is to count the number of successes among patients receiving the surgery. But this will not suffice, because the surgery is “good” or “bad” not in a vacuum but rather relative to the lack of the surgery. So the evaluation must necessarily be comparative, and the real question is whether or not one is better off with the surgery.

So we need a *control* group, and we need to compare the experiences of those with the surgery to the experiences of those without the surgery. The most obvious way to do this is to simply compare the outcomes of those electing to receive the surgery to those electing not to receive the surgery. But this comparison is still problematic, because it confounds the surgery itself with the underlying differences in severity of disease that would lead one to elect the surgery. It is not unreasonable to suppose that those patients who choose the surgery are more in need of it, so the groups being compared would not be comparable. Finding a difference in outcomes would not lead to the desired conclusion, because this could be reflective of either the surgery itself or underlying differences across the comparison groups at baseline. These underlying differences would include not only the severity of the disease (or injury) but also potentially other health-seeking behaviours, which could also differ across groups.

So the solution is to *randomize* patients to the two groups, thereby ensuring that the two groups are comparable to start with, and that any differences observed at the end are due exclusively to the surgery itself. This is the objective of randomization, but unfortunately, randomization by itself falls short of attaining this rather lofty goal. As we saw, the use of permuted blocks within the context of a randomized clinical trial can lead to prediction of future allocations, which in turn can allow for a selection bias that creates systematically different treatment groups (i.e., one group will be healthier overall than the other). So the solution to this problem is *allocation concealment*, meaning the inability for the investigator to predict upcoming allocations. This would involve, among other steps, the use of a better randomization procedure than permuted blocks. Perhaps the maximal procedure (Berger, 2005) would be used instead, to control the ability of investigators to predict upcoming allocations. But without masking, there is still the potential for patients in one group to be rated differently than those in the other group.

Many endpoints are subjective, and are not measured but rather are rated, or scored. If the rater or scorer is aware of the treatment received by the patient being rated or scored, then the treatment group could influence the rating. That is, even two identical patients may receive different ratings based only on the fact that one received the surgery and one did not. Moreover, this could be a problem even for objective endpoints, including mortality, because patients in one treatment group may receive more ancillary care and/or attention than patients in the other group, and this could lead to better outcomes for the one group, even when these outcomes are measured objectively. The solution to this problem is *masking*, or concealing the identities of the treatments for each patient until all the data are collected.

Control groups, randomization, allocation concealment, and masking are all tools of the biostatistician, and all help in assisting intuition where it otherwise could go wrong. One could argue that the effort to develop tools such as these, to assist our intuition, has been going on since well before the term “biostatistics” was coined. Yet today we may still refer to all of these activities, in aggregate, as the essence of biostatistics.

Whether conducted by biostatisticians *per se* or by others, biostatistics remains crucial. Medical research is too important for any medical researcher not to function as a biostatistician, at least to some extent.

## Implications For The Future

The set of methods discussed in the last section, specifically control groups, randomization, allocation concealment, and masking, form a collection of mutually supporting strategies to minimize bias. The residual bias that remains tends to diminish with the introduction of each additional method. It is important to note that even with all of these methods operating in conjunction, some residual bias may still remain. This means that even now, biostatistics must not be viewed as a static enterprise. Rather, it needs to be an ongoing process of trying to improve the inferences that we make from data, because even the currently best set of statistical methods sometimes generate results that are (objectively) “wrong”. This is because it is not possible to enumerate every conceivable type of bias that could occur. But the impossibility of complete success should not be taken as an indication of the futility of the exercise. By identifying biases and correcting for them on an ongoing basis, we can make studies better and better. Hence, the “best set of methods” needs to constantly evolve.

## Null Hypothesis, Type I and Type II Errors

When looking at data, biostatisticians start from the assumption that “nothing is going on here,” and these observations occurred randomly, or just by chance. This position is known as the *null hypothesis*. Then, biostatisticians ask, “How likely would this observed pattern in the data be, given that the null hypothesis is true?” If they conclude that there is a very small probability that the state of affairs observed could have occurred by chance, then the null hypothesis is rejected in favour of an alternative hypothesis. However, sometimes rare events do occur by chance, which cause researchers to reject the null hypothesis even when the null hypothesis is true; this is called Type I error. Conversely, researchers sometimes conclude that they cannot reject the null hypothesis even though an alternative hypothesis is true; this is called Type II error. By returning to the coin-flipping example, we can see how these types of error can occur. Assuming that a fair coin is being used, the probability of heads on any given flip is 0.5. Because each coin flip is independent, the probability of two successive heads would be 0.5*0.5=0.25; the probability of three successive heads would be 0.5*0.5*0.5=0.125; and the probability of four successive heads would be 0.5*0.5*0.5*0.5=0.0625. In other words, the probability of a fair coin coming up heads four times successively is rather low, which means that a research question could be formulated as the following decision rule: we will assume that this coin is fair unless, in four successive flips, the coin always comes up heads.

Under this scenario, the null hypothesis is that the coin is fair and the outcome of the four successive flips would be the data that would be marshaled to test whether the null hypothesis should be rejected. However 0.0625 is not zero, which means that, even with a fair coin, the rare event of four successive heads can occur, which would cause the researcher to reject a true null hypothesis (Type I error). Alternatively, a coin can be unfair because it has been weighted to come up heads more frequently than tails. However, as long as the coin does occasionally come up tails, there might not be four successive heads in a row, which would cause the researcher not to reject the claim that the coin is fair—even though this claim is false (Type II error). With more and more tosses, it is possible to make the error rates smaller and smaller.

As we have seen, biases may make it likely that the wrong conclusion will be reached. If two treatments are equally effective, so that the null hypothesis of their equality is true, then the wrong conclusion could be reached for either of two reasons. This could be a rare occurrence (by chance, the healthier patients all ended up in one treatment group and artificially made that treatment look better than it really is), or it could be a bias (the healthier patients were differentially recruited to one group).

## Outcome And Process

So, statistical techniques do not offer us absolute certainty. Nevertheless, there is still a connection between statistical methods and objectivity. To see this, it is important to draw a distinction between *outcome* and *process* in thinking about biostatistics. As the coin-flipping example illustrates, it is quite true that a particular statistical test may lead to the wrong conclusion in a particular instance. The data themselves may mislead due to an unfortunate sample that fails to reflect the reality of the population from which it is drawn. Good design aims to make such misleading data unlikely, but not impossible. And certainly rare events do occur. But this fact does not invalidate the *procedural* correctness of statistical techniques, in general, provided (and this is an important stipulation) that the most appropriate statistical tests are performed given the data at hand. This means that professional statisticians should be consulted in all phases of a clinical trial—from design, to implementation, to interpreting the results.

Biostatistics is an ongoing process to improve the quality of scientific inference. If one becomes aware of a bias, and of a way to correct for it, then one must do so, even if unbeknownst to the researcher, there was another bias that already compensated for the identified one. As the examples of Type I and Type II error illustrate, doing the right thing may lead to the wrong result. But one still must do the right thing because one would never know if the result is right or wrong in any given situation. While we cannot say that any particular conclusion is “right,” what we can say is that the right procedures are defined as “right” because they lead to less biased conclusions more often than other procedures would. This attempt to minimize the probability of error is about all one can hope for in scientific inference.

In the endeavour to produce methods that will in turn produce correct results, the progress that we, as a discipline, have made so far, though substantial, remains but a drop in the ocean compared to the work yet to be done. While words such as “rigorous” are thrown around with regularity, the fact is that almost all medical studies – even randomized clinical trials – are susceptible to biases that offer a compelling competing explanation for apparent treatment effects. That is, even with modern designs and analyses, results still cannot generally be accepted at face value. One must distinguish between biases that are preventable and those that are not. This line of demarcation varies with time, as one effort of biostatistics is to expand the set of biases that can be prevented. But at any one point in time, there will be a set of biases that can be prevented, and inevitably there will be many studies that make no effort to prevent them.

For example, the first classic challenge to intuition discussed above related to long runs of heads in tosses of a fair coin, and this may occur in blocked randomized trials due to a lack of allocation concealment. The resulting selection bias could lead to the spurious conclusion that the active treatment is effective when in fact it is not. This bias can be prevented by using randomization methods other than permuted blocks (Berger, 2005a; Berger, Ivanova and Deloria-Knoll, 2003), so as to help ensure allocation concealment. But in practice this is rarely done, in part because the initial definition of “allocation concealment” was grossly inadequate, and dealt with only direct observation of allocations to be made, as opposed to their prediction through the patterns in the allocation sequence that arguably constitute the greater threat (Berger, 2005b). Many researchers incorrectly believe that the methods aimed at preventing only the direct observation of the allocation sequence and assignments in sealed envelopes suffice to ensure allocation concealment, and so they make no further effort to improve the overall process. This error in judgment is what allows trials to lack allocation concealment and for the resulting selection bias to remain undetected.

Clearly, a current trial without allocation concealment is no more susceptible to selection bias than is an older trial conducted prior to the identification of allocation concealment. And yet the quality of the two studies should not be treated interchangeably. There are elements that affect trial quality and reliability that can never be checked, and investigators have discretion in either doing the best job possible or not. We would like to give the benefit of doubt to the investigators, and honest mistakes allow us to validly do so. It is not ideal to allow for a bias in a study even if that bias has yet to be discovered, but this would be an unavoidable error that does not call into question the integrity of the entire undertaking. In contrast, conducting a study that allows for a bias that is both known and preventable does not permit so favourable a view.

This discussion illustrates the important activities in which biostatisticians may engage, including:

1. Identifying biases;
2. Identifying methods to prevent, detect, and correct for these biases;and
3. Arguing for using these specialized methods as the situation warrants.

Clearly, all the efforts go to waste if the methods developed are not used when they should be. These three activities encapsulate the meaning of “biostatistics” for us. The activities involve the curiosity to ask:

What can go wrong even when everything seems to be right?

It also involves the mathematical training to develop methods to address whatever biases may be found, and the willingness to then fight the good fight against inertia and the reluctance to deviate from precedent.

## Concluding Remarks

Many researchers view biostatistics as merely an annoyance, only for satisfying journal editors, regulatory authorities, or funding agencies. But the necessity of biostatistics is apparent when considering the relationship between our intuitions and formal analytical methods. Laplace observed that the theory of probability is:

…at bottom only common sense reduced to calculus; it makes us appreciate with exactitude that which exact minds feel by a sort of instinct without being able ofttimes to give a reason for it. It leaves no arbitrariness in the choice of opinions and sides to be taken; and by its use can always be determined the most advantageous choice (Laplace, 1951).

The implication is that few of us have sufficiently “exact minds” to always intuit the correct answer—which is where analytical decision tools come into play. Given this, is there a sense in which intuition can combine with biostatistical methodology to improve understanding? The answer depends on the context in which the question is asked. As Reichenbach noted (1938), there is a “context of discovery” and a “context of justification.” That is, when a researcher is formulating a hypothesis (the “context of discovery”), a researcher can draw on any number of insights—intuition, the opinion of esteemed colleagues, peer-reviewed journal articles etc.—in formulating the hypothesis to be tested. However, when the researcher must *justify* the conclusion reached while testing that hypothesis (the “context of justification”), the appropriate biostatistical methodology rather than intuition must be relied on. In such a situation, not using proper biostatistical principles is rushing to obtain answers to important questions without bothering to ensure the integrity or accuracy of these answers. This would be akin to shunning a map on the basis of being a driver, and not a navigator. Yes, you will still arrive at a destination, but that destination may not be the one for which you set out. When driving to the wrong location, this fact becomes immediately apparent upon arriving there. In medical studies, the ability to check obtained results against the truth is never available, so the only recourse is to become the best navigator possible.

Only by navigating towards the truth can we hope to find the truth. Because our intuition often can be wrong *even when steps are taken to address this very problem*, this lesson has to be learned anew by each new generation of researchers. The process of continually trying to improve on past methods is what “biostatistics” means to us.

## Questions That This Paper Raises

1. Given our current state of knowledge, what are the known and preventable biases that should be controlled for in designing clinical trials?
2. From a broader societal perspective, why would the failure to try continually to improve on past methods in research design be ethically problematic?
3. What is the danger in assuming that we know more than we actually do?
4. What biases cannot be prevented in medical research?
5. In any given study, are there other biases, not yet identified, that could interfere with validity?

## About The Authors


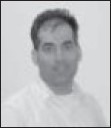


**Dr. Berger** holds degrees in statistics from Cornell University, Stanford University, and Rutgers University. His professional career has included work in the pharmaceutical industry (Janssen Research Foundation, Theradex, and some consulting for Pfizer), work in two centers of the Food and Drug Administration (Drugs and Biologics), and he now serves as a Mathematical Statistician for the National Cancer Institute. An active researcher, Dr. Berger recently wrote a book on the design and analysis of randomized clinical trials, has also authored numerous book chapters and scientific articles appearing in the peer-reviewed literature, and has presented numerous invited lectures on this topic. Dr. Berger is the recipient of the Gertrude Cox Award and an elected member of the International Statistical Institute. Dr. Berger reviews manuscripts for a number of statistical and medical journals.


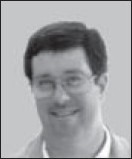


**Dr. Matthews** received his undergraduate degree in mathematics and philosophy from the College of William and Mary and earned a Ph.D. in the history of science from Duke University. He has taught at a number of institutions—including the University of Oklahoma, the University of Wisconsin-Madison, Virginia Tech and was a DeWitt Stetten, Jr. Memorial Fellow in the History of Biomedical Sciences and Technology at the National Institutes of Health. He is the author of the scholarly book Quantification and the Quest for Medical Certainty, which traces the history of biostatistical methods from the late 18^th^ century up to the emergence of randomized clinical trials in the mid 20^th^ century. He has also contributed biographical and historical articles on biostatistics in encyclopedia and reference works. He is now completing a Master of Public Health degree at Virginia Commonwealth University.
